# Parasitic infections related to anti-type 2 immunity monoclonal antibodies: a disproportionality analysis in the food and drug administration’s adverse event reporting system (FAERS)

**DOI:** 10.3389/fphar.2023.1276340

**Published:** 2023-11-14

**Authors:** Victor Pera, Guy G. Brusselle, Sebastian Riemann, Jan A. Kors, Erik M. Van Mulligen, Rowan Parry, Marcel de Wilde, Peter R. Rijnbeek, Katia M. C. Verhamme

**Affiliations:** ^1^ Department of Medical Informatics, Erasmus University Medical Center, Rotterdam, Netherlands; ^2^ Department of Respiratory Medicine, Ghent University Hospital, Ghent, Belgium; ^3^ Departments of Epidemiology and Respiratory Medicine, Erasmus University Medical Center, Rotterdam, Netherlands

**Keywords:** biologicals, monoclonal antibodies, disproportionality analysis, parasitic infections, spontaneous reporting, FAERS, pharmacovigilance, helminth infections

## Abstract

**Introduction:** Monoclonal antibodies (mAbs) targeting immunoglobulin E (IgE) [omalizumab], type 2 (T2) cytokine interleukin (IL) 5 [mepolizumab, reslizumab], IL-4 Receptor (R) α [dupilumab], and IL-5R [benralizumab]), improve quality of life in patients with T2-driven inflammatory diseases. However, there is a concern for an increased risk of helminth infections. The aim was to explore safety signals of parasitic infections for omalizumab, mepolizumab, reslizumab, dupilumab, and benralizumab.

**Methods:** Spontaneous reports were used from the Food and Drug Administration’s Adverse Event Reporting System (FAERS) database from 2004 to 2021. Parasitic infections were defined as any type of parasitic infection term obtained from the Standardised Medical Dictionary for Regulatory Activities^®^ (MedDRA^®^). Safety signal strength was assessed by the Reporting Odds Ratio (ROR).

**Results:** 15,502,908 reports were eligible for analysis. Amongst 175,888 reports for omalizumab, mepolizumab, reslizumab, dupilumab, and benralizumab, there were 79 reports on parasitic infections. Median age was 55 years (interquartile range 24–63 years) and 59.5% were female. Indications were known in 26 (32.9%) reports; 14 (53.8%) biologicals were reportedly prescribed for asthma, 8 (30.7%) for various types of dermatitis, and 2 (7.6%) for urticaria. A safety signal was observed for each biological, except for reslizumab (due to lack of power), with the strongest signal attributed to benralizumab (ROR = 15.7, 95% Confidence Interval: 8.4–29.3).

**Conclusion:** Parasitic infections were disproportionately reported for mAbs targeting IgE, T2 cytokines, or T2 cytokine receptors. While the number of adverse event reports on parasitic infections in the database was relatively low, resulting safety signals were disproportionate and warrant further investigation.

## Introduction

The discovery of human immunoglobulin E (IgE) in 1968 (Bennich et al., 1968) and an increased understanding of type 2 (T2) inflammatory pathways since the 1990s contributed to the development of today’s monoclonal antibodies (mAbs) targeted at T2 inflammation driven diseases (Fahy, 2015). Within asthma there is an endotype that is broadly characterized by T2 inflammation, namely, T2 asthma (Kuruvilla et al., 2019). T2 asthma demands a different treatment strategy than non-T2 asthma. Besides T2 asthma, also chronic urticaria, chronic rhinosinusitis with nasal polyps (CRSwNP), and atopic dermatitis are characterized by T2 inflammation (Garcovich et al., 2021; Matucci et al., 2021). While increased blood eosinophils are a biomarker for T2 asthma, a differential diagnosis is extensive and includes CRSwNP, vasculitis, and parasitic disease (Piggott et al., 2022). These eosinophils contribute to innate immune responses against helminths (i.e., multicellular parasitic worms) through phagocytosis, release of cytotoxic proteins and formation of extracellular traps (Klion, et al., 2020).

The availability of biologicals has aided patients with severe asthma in reducing exacerbations and oral corticosteroid use, while improving lung function and quality of life, especially in patients with T2 asthma (McGregor et al., 2019). The IgE-binding mAb omalizumab was approved in 2002 for the treatment of moderate-to-severe allergic asthma among adults and adolescents by the Therapeutic Goods Administration in Australia (BioDrugs, 2002). After that, more mAbs targeting T2 asthma were approved, namely, mepolizumab, reslizumab, benralizumab, and dupilumab (Papi et al., 2020). Dupilumab was primarily registered for the treatment of moderate-to-severe atopic dermatitis in 2017 (Shirley, 2017), for the treatment of asthma in 2018, and for the treatment of CRSwNP in 2019 (Boyle et al., 2020). Omalizumab was additionally registered for the treatment of chronic urticaria in 2014 (Kaplan et al., 2017). Clinical trials have shown that these biologicals contribute to disease control of T2 inflammatory diseases, especially in patients with T2 asthma (Agache et al., 2021; Matucci et al., 2021). While the effectiveness of these biologicals in real life has been demonstrated, further evaluation of the long-term safety of biologicals is needed as safety profiling studies are limited (Brusselle and Koppelman, 2022). In the clinical trials safety profiles were similar for patients in the intervention group and placebo group, with a low number of serious adverse events (AEs) (Ortega et al., 2014; Bleecker et al., 2016; Castro et al., 2018). More recently post-marketing studies confirm the low incidence of serious AEs in patients receiving anti-T2 biologicals, while identifying previously unknown risks (Sousa et al., 2020; Bettuzzi et al., 2022; Galletti et al., 2023). Anaphylaxis signals have been described for omalizumab, benralizumab, reslizumab, and mepolizumab (Li et al., 2021). Dupilmab has been linked with eye disorders, especially in patients with atopic dermatitis (Park et al., 2021).

In recent years, concerns were expressed for a hypothesized increased risk of parasitic infections among patients using biologicals affecting the T2 immune response (Tan et al., 2019). Such biologicals are dupilumab, omalizumab, mepolizumab, benralizumab, and reslizumab. Omalizumab binds to free IgE, inhibiting further binding of IgE to high-affinity IgE receptors on mast cells and basophils (Brusselle and Koppelman, 2022), while IgE is considered an important part of the multi-component T2 immune response towards parasitic infections (Cooper et al., 2008; Fitzsimmons et al., 2014). Dupilumab binds to interleukin (IL-) 4 receptor α, which inhibits IL-4 and IL-13 signaling (Brusselle and Koppelman, 2022), while both cytokines contribute to the elimination of parasites through increased mucus production and eosinophilic mucosal inflammation in the gut (Klion and Nutman, 2004; Braddock et al., 2018). Benralizumab binds to IL-5Rα, resulting into depletion of eosinophils in the blood and mucosal tissues via antibody-dependent cell-mediated cytotoxicity (Brusselle and Koppelman, 2022), and might thus interfere with the eosinophil-mediated killing and expulsion of gastro-intestinal helminths (Klion and Nutman, 2004). Reslizumab and mepolizumab bind to circulating interleukin IL-5 (Brusselle and Koppelman, 2022), likewise an important cytokine contributing to eosinophilic differentiation and activation (Klion and Nutman, 2004; Maizels and Adam, 2004).

In current clinical studies and routine practice, reporting of parasitic infections is based on spontaneous reporting, rather than systematic anamnestic or biochemical screening. Especially in endemic regions, routine screening for parasitic infections may be useful. So far, no studies were performed on the Food and Drug Administration’s (FDA) Adverse Event Reporting System (FAERS) investigating the risk of parasitic infections among patients using biologicals affecting the T2 immune response. Therefore, the objective of this study was to determine if parasitic infections are disproportionately reported for biologicals affecting the T2 immune response.

## Methodology

### Data source

For this study, we used publicly available quarterly data files from FAERS covering the period 2004–2021 (Food and Drug Administration, 2021; FAIRsharing Team, 2022). FAERS data represents data from spontaneous reports on (product quality complaints resulting in) AEs and medication errors in relation to medication (excluding vaccines) and medical devices (Questions and Answers on FDA’s Adverse Event Reporting System (FAERS), 2019). The FDA receives reports from healthcare professionals, consumers, manufacturers, and lawyers. The FDA’s MedWatch Online Voluntary Reporting Form is freely available to the worldwide public and facilitates sending in a report (MedWatch Online Voluntary Reporting Form, 2019). Preferred Terms (PTs) from the Medical Dictionary for Regulatory Activities^®^ (MedDRA^®^) are used as a standardized coding practice for the reported events in a received report.

### Data processing

Data processing of the quarterly files was performed by the Adverse Event Open Learning through Universal Standardization (AEOLUS) system (Parry, 2021a). AEOLUS performs standardization of the FAERS data, case deduplication, and disproportionality analyses as described in further detail by Banda et al. (2016). The algorithm removes any duplicate cases based on exact matches on the combined demographic fields, list of drugs and list of outcomes (FAERS reactions). Additionally, the FDA and manufacturers provide a list of suggested cases to be deleted within each batch of quarterly datafiles since the first quartile of 2019 for various purposes, including combining cases. The original AEOLUS scripts were adapted for downloading and processing data up to the final quarter of 2021. Mapping of drug names to the Anatomical Therapeutic Chemical (ATC) coding system was performed by the locally developed AIOLI system (Parry, 2021b). Only drugs mapped to an ATC code and marked as primary suspect within a FAERS report were included in the analysis. All age units in the data were converted to years. The age variable was marked as invalid in case of a negative value, no indicated age unit, or missing age.

### Case definition and selection process

All the selected FAERS reports from the FAERS database as described previously were processed. The reported AE of interest was a parasitic infection, which was defined as any PT falling under the MedDRA^®^ High Level Group Term (HLGT) “Helminthic Disorders” or a PT related to parasitic infections noted under High Level Term (HLT) “Infections—Not elsewhere classified (NEC)” according to MedDRA^®^ version 24.1. All these PTs are listed in Supplementary Table S1. Descriptive statistics and disproportionality results were presented for reports stating a parasitic infection and one of the following mAbs marked as the primary drug suspect: dupilumab (ATC: D11AH05), omalizumab (ATC: R03DX05), mepolizumab (ATC: R03DX09), benralizumab (ATC: R03DX10), and reslizumab (ATC: R03DX08).

### Disproportionality analyses

Signals of disproportionate reporting (SDRs) were produced by AEOLUS and expressed as Reporting Odds Ratio’s (RORs) with a 95% confidence interval (CI). The ROR and 95% CI were calculated according to Eqs 1, 2. Letters A, B, C, and D in Eqs 1, 2 represent drug-event combinations (DECs) as specified in Table 1.

$$ROR=\frac{A/C}{B/D}$$ (1)

$$95\%\;CI\;for\;ROR=e^{\ln\left(ROR\right)\;\pm 1.96\sqrt{\frac{1}{A}+\frac{1}{B}+\frac{1}{C}+\frac{1}{D}}}$$ (2)

**TABLE 1:** Overview of report counts based on various drug-event combinations (DECs) expressed by the letters A, B, C, and D, including variations on counts C and D, represented by the dagger (†) and double dagger (‡), for the purpose of various disproportionality analyses as previously represented by Eqs 1, 2 in the manuscript. The found fixed A and B counts for every biologic of interest were used in every analyses, while the C and D counts were different per analysis.

	Parasitic infection	Other events	Target analysis
A biologic of interest	A	B	All analyses
All other drugs	C	D	Primary analysis
All other drugs, excluding ATC group P drugs	C †	D †	1st secondary analysis
All other biologics of interest	C ‡	D ‡	2nd secondary analysis

ATC, anatomical therapeutic chemical classification, group P, antiparasitic products, insecticides and repellents.

A SDR was considered disproportionate if the lower boundary of the 95% CI was greater than 1 and the drug-event count was at least 3, in accordance to guidelines from the European Medicines Agency (EMA) (European Medicines Agency, 2016). Three disproportionality analyses were performed as described in Table 1: i) using DECs over the entire FAERS database, ii) using DECs over the entire FAERS database excluding drugs under ATC group P [antiparasitic products, insecticides, and repellents] and iii) using DECs of only dupilumab, omalizumab, mepolizumab, benralizumab, and reslizumab. Descriptive statistics were performed on the reports relating to parasitic infections and users of the mAb therapies of interest.

## Results

### Primary analysis

**FIGURE 1:**
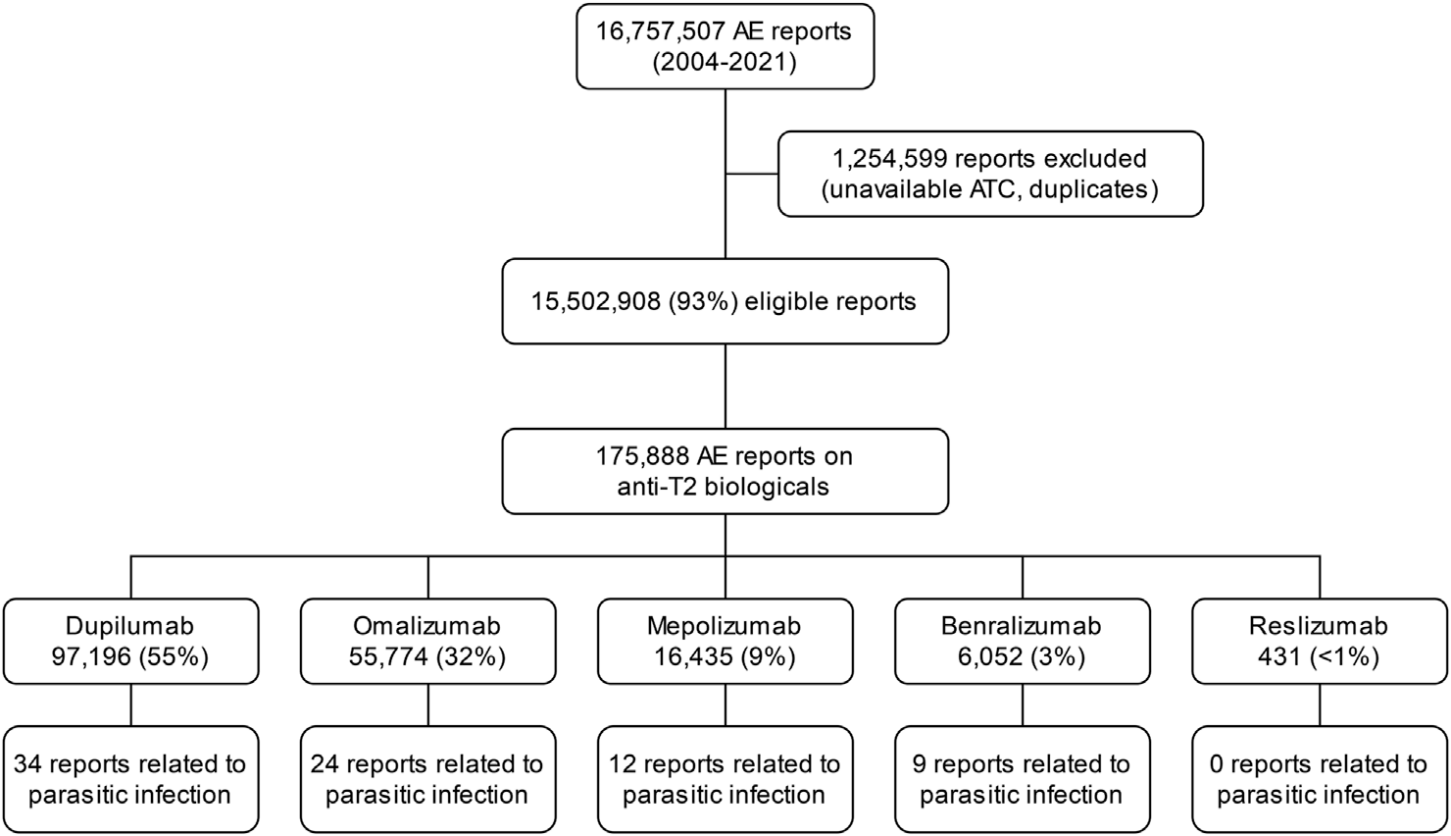
Overview of the data extraction process.

The FAERS database contains 16,757,507 AE reports from 2004 to 2021. The data extraction process is displayed with a flowchart (Figure 1). A total of 15,502,908 (93%) reports were eligible for the primary disproportionality analysis based on the availability of an ATC code for the primary drug suspect. Among the 175,888 AE reports concerning anti-T2 immunity biologicals in FAERS, 97,196 (55%) were on dupilumab, followed by 55,774 (32%) for omalizumab, 16,435 (9%) for mepolizumab, 6,052 (3%) for benralizumab, and 431 (<1%) for reslizumab. For these biologicals, 79 reports on parasitic infections were found within FAERS, mentioning 81 PTs related to parasitic infections. The following report characteristics are displayed in Table 2. Reported median age within the 79 reports was 55 years with an interquartile range of 24–63 years. Most of the reports indicated sex [47 (59.5%) individuals were female]. The reports were mostly submitted by consumers, accounting for 44 (55.7%) reports, followed by 25 (32.9%) submitted by physicians. Most reports originated from the United States of America (USA), being 58 (73.4%). Indications were mentioned in 26 reports; 14 (53.8%) biologicals were reportedly prescribed for asthma, 5 (19.2%) for atopic dermatitis and 2 (7.7%) for dermatitis. The following indications were reported only once: “chronic spontaneous urticaria,” “eosinophil count increased,” “nasal polyps,” “neurodermatitis,” and “urticaria chronic.” FAERS case numbers and data on case level for these reports can be found in Supplementary Table S2.

**TABLE 2:** Characteristics of reports related to the monoclonal antibodies of interest with a mention of parasitic infection.

Total reports of interest	79
Median Age, in years (IQR)	55 (24–63)
	Count (proportion)
Sex	
Female	47 (59.5%)
Male	18 (22.8%)
Unknown	14 (17.7%)
Reporter type	
Consumer	44 (55.7%)
Physician	26 (32.9%)
Pharmacist	1 (1.3%)
Other health-professional	5 (6.3%)
Unknown	3 (3.8%)
Reporter country	
United States	58 (73.4%)
15 other countries, each contributing <5%	21 (26.6%)
Indication	
Asthma	14 (17.7%)
Dermatitis atopic	5 (6.3%)
Dermatitis	2 (2.5%)
Chronic spontaneous urticaria	1 (1.3%)
Eosinophil count increased	1 (1.3%)
Nasal polyps	1 (1.3%)
Neurodermatitis	1 (1.3%)
Urticaria chronic	1 (1.3%)
Unknown	53 (67.1%)

IQR, interquartile range.

**FIGURE 2:**
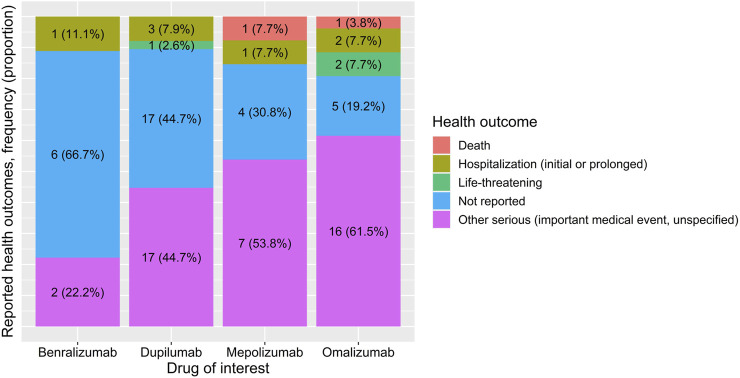
Reported health outcomes for benralizumab, dupilumab, mepolizumab, and omalizumab, expressed in absolute numbers and proportions per drug.

Health outcomes varied per mAb of interest (Figure 2). Omalizumab reports had proportionally the most reported health outcomes and the least missing outcomes. The most commonly reported outcome was “other serious (important medical event, unspecified),” accounting for 16 (61.5%) of the omalizumab reports. The highest proportion for no health outcome reported was for benralizumab, being 6 (66.7%) reports. Death has been reported in 1 (7.7%) report for mepolizumab and 1 (3.8%) in omalizumab. The “life-threatening” health outcome was reported in 2 (7.7%) reports of omalizumab and 1 (2.6%) report of dupilumab. Available raw data on start date therapy, end date therapy, and time of event were provided in Supplementary Table S3.

**FIGURE 3:**
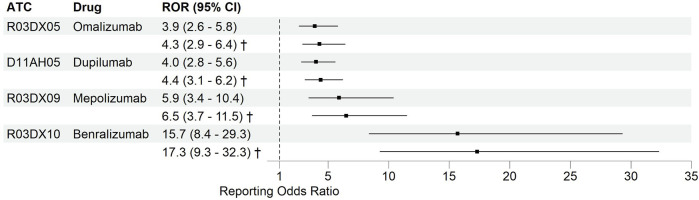
Signals of disproportionate reporting for biologics of interest compared to other drugs concerning parasitic infections. Results are shown of the primary analysis utilizing the entire database and of the 1st secondary analysis. No signal could be produced for reslizumab due to lack of power. † Results from the 1st secondary analysis utilizing the entire database, excluding drugs under Anatomical Therapeutic Chemical classification group Antiparasitic products, insecticides and repellents [see methods for detailed description]. ATC, Anatomical Therapeutical Chemical classification; CI, Confidence Interval; ROR, Reporting Odds Ratio.

Parasitic infections were found in 34 (43%) reports for dupilumab, followed by 24 (30.4%) reports for omalizumab, 9 (11.4%) for benralizumab, and 12 (15.2%) for mepolizumab. No parasitic infections were reported for reslizumab. The specific parasitic infections for each biological can be found in Supplementary Table S2. Benralizumab showed the strongest SDR for the primary analysis (Figure 3), with a ROR of 15.7 (95% CI: 8.4–29.3). The second strongest SDR was for mepolizumab, showing a ROR of 5.9 (95% CI: 3.4–10.4). The SDRs for omalizumab and dupilumab were similar, with RORs of 3.9 (95% CI: 2.6–5.8) and 4 (95% CI: 2.8–5.6), respectively. For reslizumab no ROR could be calculated in any analysis because there were no AE reports on parasitic infections for this biological.

### Secondary sensitivity analyses

**FIGURE 4:**
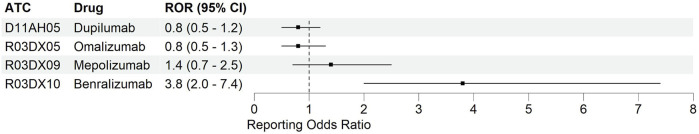
Signals of disproportionate reporting within the group of biologics of interest concerning parasitic infections. Results are shown of the 2nd secondary analysis utilizing reports only from dupilumab, omalizumab, mepolizumab, benralizumab, and reslizumab [see methods for detailed description]. No signal could be produced for reslizumab due to lack of power panel. ATC, Anatomical Therapeutical Chemical classification; CI, Confidence Interval; ROR, Reporting Odds Ratio.

In the 1st secondary analysis [Figure 3, results marked by a dagger (†)], we excluded 30,748 AE reports concerning drugs under ATC level 5 group P [Antiparasitic products, insecticides and repellents], to minimize the risk of bias (e.g., due to “reverse causation”). In this secondary analysis, the SDRs for the biologicals of interest were similar to those in the primary analyses, however with a slight increase in ROR for all biologicals. In the 2nd secondary analysis, we tested the disproportionality on parasitic infections only within the group of selected biologicals (Figure 4). Parasitic infections were disproportionately reported for benralizumab compared to the other biologicals of interest. The ROR for benralizumab was 3.8 (95% CI: 2–7.4), while the RORs for dupilumab, omalizumab, and mepolizumab were not signifying disproportionality. Details of various DECs are illustrated in Supplementary Tables S4–S6 in the Online Repository.

## Discussion

Even though a limited amount of AE case reports on parasitic infections were retrieved from the FAERS database, we demonstrated SDRs for parasitic infections associated with anti-IgE omalizumab and anti-T2 cytokine (receptor) antibodies dupilumab, mepolizumab, and benralizumab. In addition, we discovered that within the group of these biologicals parasitic infections were disproportionately reported for benralizumab. Given the mechanism of action of benralizumab [binding to IL-5R on eosinophils and basophils, and consequently depleting eosinophils in the blood and mucosal tissues such as the gastro-intestinal tract through antibody-dependent, cell-mediated toxicity] (Brusselle and Koppelman, 2022), it could be reasoned why proportionally more AE reports on parasitic infections were found in FAERS for benralizumab compared to the other biologicals. These SDRs should be taken seriously since a major clinical impact has been reported in the case reports, including death, life-threatening situations, hospitalizations, and other serious (unspecified) medical events.

For reslizumab no AE reports were found on parasitic infections within FAERS, which could be attributed to a general low reporting rate for this biological and most probably due to lack of power. Indeed, spontaneous reports for the other biologicals were roughly a 12–16-fold more often received by the FDA than for reslizumab. The lower reporting rate for reslizumab might be due to a low number of patients receiving the drug because reslizumab has to be administered intravenously in the hospital. In contrast, other biologicals can be self-administered subcutaneously (e.g., at home), making treatment with reslizumab less practical and potentially more costly within the healthcare system (Kavanagh et al., 2021). Excluding drugs from the ATC drug group P in the 1st secondary analysis showed a slight increase in disproportionality for all the biologicals of interest, indicating a confounding effect.

Notably, 50 out of 79 parasitic infections were unspecified, raising concerns on the validity of the submitted reports. The parasitic infections might have been reported by individuals, such as consumers or even healthcare workers, with no experience in the diagnosis of parasitic infections. On the other hand, sufficient information might have been provided by an experienced or treating healthcare professional to the reporter of the AE. The validity of the start date of treatment, end date of treatment, and event date (i.e., date of parasitic infection) should be carefully interpreted, as in most cases these data elements were not reported, and in some cases the parasitic infection was reported before the start date of the mAb treatment. The latter would be unlikely as only primary drug suspects for the reported events were selected for analysis. Recently, a correspondence article by Lifar et al. (2023) has been published describing a disproportionality analysis of parasitic infections among omalizumab, mepolizumab, benralizumab, and dupilumab within the VigiBase using a case/noncase design in which the control group was represented by the disease concept “asthma” in combination with at the time approved inhalation therapies. Lifar et al. (2023) showed that only benralizumab among the biologicals of interest was disproportionately reported for parasitic infections compared to the control group. In comparison, our study showed disproportionality for all biologicals of interest, except for reslizumab, while utilizing the entire FAERS database representing a broader range of patients and medication.

While theoretically it can be expected that patients using these biologicals would be at an increased risk for parasitic infections, literature is relatively scarce on the topic, and evidence on the increased risk is weak. A 2007 clinical trial performed in Brazil reported that 50% (34 of 68) of the omalizumab arm experienced at least 1 intestinal helminth infection, compared to 41% (28 of 69) of the placebo arm (Cruz et al., 2007). The odds ratio (OR) was 1.47 with a 95% CI of 0.74–2.95. The OR was 2.2 (95% CI: 0.94–5.15) after adjusting for study visit, baseline infection status, sex, and age. In a 2013 omalizumab study in Turkey with 19 participants having severe asthma, 1 case of giardiasis was reported (Yalcin et al., 2013). An observational Italian study between 2007 and 2016 in which 91 patients received omalizumab did not report any parasitic infections (Di Bona et al., 2017). A 2021 clinical trial showed that 7 out of 271 (2.6%) children with uncontrolled moderate-to-severe asthma in the dupilumab arm experienced a non-severe parasitic infection compared to 0 out of 134 subjects in the placebo arm (Bacharier et al., 2021). A 2019 pooled analysis on infections in 1841 atopic dermatitis patients using dupilumab showed that no parasitic infections were reported (Eichenfield et al., 2019). In a review from 2019 by Tan et al. (2019) no cases of parasitic infections were reported in clinical trials assessing dupilumab, benralizumab, reslizumab, and mepolizumab in patients with severe asthma. A more recent 2021 review by Dragonieri and Carpagnano highlighted additional studies for mepolizumab and reslizumab, however, did not find reported cases with parasitic infections (Dragonieri and Giovanna Elisiana, 2021). In all of the summary of product characteristics (SmPCs) of the studied biologicals, it is recognized that IgE and eosinophils are involved in the immunological response for parasitic infections, hence some caution should be considered when individuals at high risk of parasitic infection are treated with the discussed biologicals (European Medicines Agency, 2015; European Medicines Agency, 2020; European Medicines Agency, 2021; European Medicines Agency 2022b; European Medicines Agency 2022a).

Besides the well-known shortcomings of randomized clinical trials (RCTs) compared to observational studies (Hannan, 2008), RCTs involving the discussed biologicals often excluded patients with a (history of) parasitic infection or a recent (or planned) visit to a country with prevalent parasitic infections (Hodsman et al., 2013; Wenzel et al., 2013; Beck et al., 2014; Braddock et al., 2018; Eichenfield et al., 2019; Paller et al., 2022). A 2022 open-label extension study on the safety and efficacy of dupilumab also excluded individuals which were suspected or at high risk of parasitic infections (Wechsler et al., 2022). Therefore, more real world studies on this topic should be performed to gain deeper understanding on the effect of anti-T2 immunity biologicals on parasitic infections.

### Limitations and strengths

While the FAERS database contains a rich dataset which is publicly available, it is subject to underreporting and selective reporting of cases (Alatawi and Hansen, 2017), potentially leading to biased results. Unmeasured and unknown confounders related to the population of the select mAb users and the population which contracted parasitic infections while being on any kind of drug therapy might have biased the results. To the best of our knowledge, there are no (strong) confounders theorized in literature which should be taken into account to produce less biased results. Potential confounding effect was addressed by excluding reports where the primary drug suspect was related to ATC group P. If the seriousness of a parasitic infection is low, then it might not be reported, as previous research among physicians showed that AEs are most probably reported in case of being a serious unknown AE of an established drug or new drug, or a serious known Adverse Drug Reaction (ADR) attributed to a new drug (Hasford et al., 2002). Even though most of the biologicals showed disproportionate reporting for parasitic infections, further root cause analysis is necessary to conclude if these biologicals were truly causative for the parasitic infections. Pharmacovigilance studies are based on spontaneous reports which allow for the calculation of RORs, however event rates cannot be calculated as these would require data on the total number of patients exposed to these biologicals. A major limitation of spontaneous reporting is indeed that causation does not have to be proven. Due to a relatively low reporting of reslizumab cases, a SDR could not be calculated. Besides underreporting, in-depth analysis of these data are hampered by missing data for the available reports, and the scarcity of demographic and clinical metadata for these cases. While dupilumab is on the market since 2002, the public FAERS database has only been made available since 2004, potentially missing more cases of parasitic infections on dupilumab. It should be noted that this is the first study, with a thoroughly described analysis and in-depth case details, performed with FAERS data on the disproportionate reporting of parasitic infections associated with biologicals targeting IgE, T2 cytokines or T2 cytokine receptors, opening up further discussions on the safety profile of these biologicals and motivating additional studies on the proposed association.

## Conclusion

Parasitic infections were disproportionately reported for mAbs targeting IgE, T2 cytokines, or T2 cytokine receptors. While the number of AE reports on parasitic infections in the FAERS database was relatively low, the resulting safety signals were disproportionate and warrant further investigation.

## Data Availability

The original contributions presented in the study are included in the article/Supplementary Material, further inquiries can be directed to the corresponding author.

## Abbreviations

ADR, Adverse drug reaction; AE, Adverse event; AEOLUS, Adverse Event Open Learning through Universal Standardization; ATC, Anatomical Therapeutic Chemical classification; CI, Confidence interval; CRSwNP, Chronic rhinosinusitis with nasal polyps; DEC, Drug-event count; EMA, European Medicines Agency; FDA, Food and Drug Administration; FAERS, FDA Adverse Event Reporting System; HLGT, High Level Group Term; HLT, High Level Term; IgE, Immunoglobulin E; IL, Interleukin; mAb, Monoclonal Antibody; MedDRA^®^, Medical Dictionary for Regulatory Activities^®^; NEC, Not elsewhere classified; OR, Odds Ratio; PT, Preferred Term; R, Receptor; RCT, Randomized clinical trials; ROR, Reporting Odds Ratio; SDR, Signal of disproportionate reporting; T2, Type 2.
