# Supplementary material for: Parasitic infections related to anti-type 2 immunity monoclonal antibodies: a disproportionality analysis in the food and drug administration’s adverse event reporting system (FAERS)
Source: Front Pharmacol. 2023 Nov 14;14:1276340. doi: 10.3389/fphar.2023.1276340 (PMC10682182; doi:10.3389/fphar.2023.1276340)
Supplement: Supplementary file 1 [file DataSheet1.docx]

**Parasitic infections related to anti-type 2 immunity monoclonal antibodies: a disproportionality analysis in the food and drug administration’s adverse event**

**reporting system (FAERS)**

Victor Pera PharmD^1*^, Guy. G. Brusselle MD, PhD^2,3^, Sebastian Riemann MD^2^, Jan A. Kors PhD^1^, Erik M. van Mulligen PhD^1^, Rowan Parry PhD^1^, Marcel de Wilde BSc^1^, Peter R. Rijnbeek PhD^1^, Katia M.C. Verhamme MD, PhD^1^

^1^ Department of Medical Informatics, Erasmus University Medical Center, Rotterdam, The Netherlands

^2^ Department of Respiratory Medicine, Ghent University Hospital, Ghent, Belgium

^3^ Departments of Epidemiology and Respiratory Medicine, Erasmus University Medical Center, Rotterdam, The Netherlands

***Correspondence**

Victor Pera

[v.pera@erasmusmc.nl](mailto:v.pera@erasmusmc.nl)

Table S1. Preferred terms from the Medical Dictionary for Regulatory Activities®, version 24.1, representing parasitic infections under the high level group term “Helminthic disorders” and “Infections - pathogen unspecified”. NEC = Not elsewhere classified

| **Code** | **Preferred Term** | **High Level Term** |
| --- | --- | --- |
| 10069517 | Angiostrongylus infection | Nematode infections |
| 10002533 | Anisakiasis | Nematode infections |
| 10060967 | Arthritis helminthic | Helminthic infections NEC |
| 10003442 | Ascariasis | Nematode infections |
| 10065200 | Biliary tract infection helminthic | Helminthic infections NEC |
| 10007187 | Capillariasis | Nematode infections |
| 10061039 | Cestode infection | Cestode infections |
| 10009344 | Clonorchiasis | Trematode infections |
| 10059547 | Cutaneous larva migrans | Nematode infections |
| 10065199 | Cystitis helminthic | Helminthic infections NEC |
| 10080485 | Dicrocoeliasis | Trematode infections |
| 10013029 | Diphyllobothriasis | Cestode infections |
| 10080486 | Dipylidiasis | Cestode infections |
| 10080290 | Dirofilariasis | Nematode infections |
| 10080496 | Disseminated strongyloidiasis | Nematode infections |
| 10013618 | Dracunculiasis | Nematode infections |
| 10014096 | Echinococciasis | Cestode infections |
| 10065327 | Endocarditis helminthic | Helminthic infections NEC |
| 10014881 | Enterobiasis | Nematode infections |
| 10065204 | Enterocolitis helminthic | Helminthic infections NEC |
| 10065180 | Eye infection helminthic | Helminthic infections NEC |
| 10016234 | Fascioliasis | Trematode infections |
| 10016235 | Fasciolopsiasis | Trematode infections |
| 10016674 | Filariasis | Nematode infections |
| 10016675 | Filariasis lymphatic | Nematode infections |
| 10065207 | Gastritis helminthic | Helminthic infections NEC |
| 10065223 | Genital infection helminthic | Helminthic infections NEC |
| 10018490 | Gnathostomiasis | Nematode infections |
| 10061201 | Helminthic infection | Helminthic infections NEC |
| 10019659 | Hepatic echinococciasis | Cestode infections |
| 10065216 | Hepatic infection helminthic | Helminthic infections NEC |
| 10020017 | Heterophyiasis | Trematode infections |
| 10020376 | Hookworm infection | Nematode infections |
| 10020546 | Hymenolepiasis | Cestode infections |
| 10021857 | Infection parasitic | Infections NEC |
| 10065210 | Lymphadenitis helminthic | Helminthic infections NEC |
| 10065171 | Meningoencephalitis helminthic | Helminthic infections NEC |
| 10027436 | Metagonimiasis | Trematode infections |
| 10065219 | Myocarditis helminthic | Helminthic infections NEC |
| 10062701 | Nematodiasis | Nematode infections |
| 10058443 | Neurocysticercosis | Cestode infections |
| 10030314 | Onchocerciasis | Nematode infections |

Table S1 continued

| **Code** | **Preferred Term** | **High Level Term** |
| --- | --- | --- |
| 10084319 | Onchodermatitis | Nematode infections |
| 10030897 | Opisthorchiasis | Trematode infections |
| 10065329 | Oral helminthic infection | Helminthic infections NEC |
| 10065189 | Pancreatitis helminthic | Helminthic infections NEC |
| 10033794 | Paragonimiasis | Trematode infections |
| 10069588 | Parasitic encephalitis | Infections NEC |
| 10067720 | Parasitic gastroenteritis | Infections NEC |
| 10056704 | Parasitic esophagitis | Infections NEC |
| 10078883 | Parasitic pneumonia | Infections NEC |
| 10065221 | Pericarditis helminthic | Helminthic infections NEC |
| 10065326 | Peritonitis helminthic | Helminthic infections NEC |
| 10065246 | Pneumonia helminthic | Helminthic infections NEC |
| 10037374 | Pulmonary echinococciasis | Cestode infections |
| 10078264 | Renal echinococciasis | Cestode infections |
| 10039603 | Schistosomiasis | Trematode infections |
| 10039605 | Schistosomiasis bladder | Trematode infections |
| 10039606 | Schistosomiasis cutaneous | Trematode infections |
| 10039610 | Schistosomiasis liver | Trematode infections |
| 10065172 | Skin infection helminthic | Helminthic infections NEC |
| 10065193 | Splenic infection helminthic | Helminthic infections NEC |
| 10042254 | Strongyloidiasis | Nematode infections |
| 10080487 | Syngamiasis | Nematode infections |
| 10043095 | Taeniasis | Cestode infections |
| 10043720 | Thyroid echinococciasis | Cestode infections |
| 10044269 | Toxocariasis | Nematode infections |
| 10061859 | Trematode infection | Trematode infections |
| 10044608 | Trichiniasis | Nematode infections |
| 10044627 | Trichostrongyliasis | Nematode infections |
| 10044630 | Trichuriasis | Nematode infections |
| 10065258 | Tropical eosinophilia | Nematode infections |
| 10065186 | Upper respiratory tract infection helminthic | Helminthic infections NEC |
| 10065328 | Vulvovaginitis helminthic | Helminthic infections NEC |
| 10065243 | Wound infection helminthic | Helminthic infections NEC |

Table S2. Reference numbers for case reports describing the use of i) omalizumab, dupilumab, mepolizumab, benralizumab or reslizumab and ii) describing a parasitic infection, accompanied by a description of indication, type of parasitic infection and year with quarter. †From the legacy adverse event reporting system database

| **Report id** | **Drug** | **Indication** | **Parasitic Infection** | **Report year** | **Quarter** | **Reporter** | **Country** |
| --- | --- | --- | --- | --- | --- | --- | --- |
| 6412861† | Omalizumab | Unknown | Infection Parasitic | 2009 | Q4 | Other health-professional | United States |
| 6422338† | Omalizumab | Unknown | Infection Parasitic | 2009 | Q4 | Physician | United States |
| 7456124† | Omalizumab | Asthma | Taeniasis | 2011 | Q2 | Physician | Italy |
| 7831160† | Omalizumab | Asthma | Helminthic Infection | 2011 | Q4 | Physician | United Kingdom |
| 88868233 | Omalizumab | Asthma | Hepatic echinococciasis | 2013 | Q2 | Other health-professional | Unknown |
| 112279541 | Omalizumab | Unknown | Infection parasitic | 2015 | Q2 | Other health-professional | United States |
| 116713721 | Omalizumab | Unknown | Infection parasitic | 2015 | Q4 | Consumer | United States |
| 120462742 | Omalizumab | Unknown | Infection parasitic | 2016 | Q1 | Physician | United States |
| 127270233 | Omalizumab | Unknown | Infection parasitic | 2017 | Q2 | Other health-professional | United States |
| 128327562 | Mepolizumab | Unknown | Infection parasitic | 2016 | Q4 | Consumer | United States |
| 130536502 | Omalizumab | Unknown | Infection parasitic | 2017 | Q1 | Physician | Canada |
| 133047542 | Omalizumab | Urticaria Chronic | Diphyllobothriasis | 2017 | Q2 | Physician | Denmark |
| 135297952 | Omalizumab | Asthma | Enterobiasis | 2017 | Q2 | Physician | United States |
| 135384151 | Omalizumab | Asthma | Strongyloidiasis | 2017 | Q2 | Physician | Spain |
| 135833931 | Omalizumab | Unknown | Infection parasitic | 2017 | Q2 | Consumer | Netherlands |
| 135951192 | Omalizumab | Chronic Spontaneous Urticaria | Opisthorchiasis | 2017 | Q3 | Physician | Russian Federation |
| 152438235 | Dupilumab | Unknown | Infection parasitic | 2018 | Q4 | Physician | United States |
| 153768121 | Omalizumab | Unknown | Infection parasitic | 2018 | Q3 | Consumer | United States |
| 154171474 | Benralizumab | Unknown | Infection parasitic | 2019 | Q2 | Physician | United States |
| 155437031 | Mepolizumab | Unknown | Strongyloidiasis | 2018 | Q4 | Other health-professional | Australia |
| 155446756 | Omalizumab | Asthma | Arthritis helminthic | 2019 | Q3 | Consumer | Brazil |
| 156369229 | Dupilumab | Dermatitis Atopic | Helminthic infection | 2020 | Q3 | Consumer | United States |
| 159035971 | Benralizumab | Asthma | Enterobiasis | 2019 | Q1 | Physician | United States |
| 159198241 | Omalizumab | Unknown | Infection parasitic | 2019 | Q1 | Physician | United States |
| 159279334 | Benralizumab | Asthma | Infection parasitic | 2019 | Q2 | Unknown | United States |
| 159279334 | Benralizumab | Asthma | Strongyloidiasis | 2019 | Q2 | Unknown | United States |
| 159385621 | Omalizumab | Unknown | Infection parasitic | 2019 | Q1 | Consumer | United States |
| 159385621 | Omalizumab | Unknown | Enterobiasis | 2019 | Q1 | Consumer | United States |
| 159389361 | Omalizumab | Unknown | Enterobiasis | 2019 | Q1 | Consumer | United States |
| 160665231 | Mepolizumab | Unknown | Infection parasitic | 2019 | Q1 | Consumer | Canada |
| 160926871 | Benralizumab | Unknown | Infection parasitic | 2019 | Q1 | Consumer | United States |
| 161733721 | Benralizumab | Asthma | Helminthic infection | 2019 | Q2 | Physician | United States |

Table S2 continued

| **Report id** | **Drug** | **Indication** | **Parasitic Infection** | **Report year** | **Quarter** | **Reporter** | **Country** |
| --- | --- | --- | --- | --- | --- | --- | --- |
| 162228012 | Mepolizumab | Unknown | Enterobiasis | 2019 | Q2 | Consumer | United States |
| 162231351 | Dupilumab | Unknown | Infection parasitic | 2019 | Q2 | Physician | United States |
| 163153155 | Dupilumab | Asthma | Enterobiasis | 2020 | Q2 | Consumer | United States |
| 164557632 | Mepolizumab | Asthma | Helminthic infection | 2019 | Q3 | Consumer | United States |
| 164821131 | Dupilumab | Unknown | Infection parasitic | 2019 | Q2 | Consumer | United States |
| 165259972 | Mepolizumab | Unknown | Infection parasitic | 2019 | Q4 | Consumer | United States |
| 165380635 | Dupilumab | Dermatitis Atopic | Helminthic infection | 2020 | Q4 | Consumer | United States |
| 165871631 | Dupilumab | Unknown | Infection parasitic | 2019 | Q3 | Consumer | United States |
| 166135231 | Omalizumab | Unknown | Infection parasitic | 2019 | Q3 | Physician | Peru |
| 166854971 | Dupilumab | Unknown | Infection parasitic | 2019 | Q3 | Consumer | United States |
| 168228951 | Mepolizumab | Unknown | Parasitic gastroenteritis | 2019 | Q3 | Consumer | Slovakia |
| 169536901 | Dupilumab | Asthma | Helminthic infection | 2019 | Q4 | Consumer | United States |
| 169750711 | Dupilumab | Unknown | Infection parasitic | 2019 | Q4 | Consumer | United States |
| 170932051 | Mepolizumab | Unknown | Strongyloidiasis | 2019 | Q4 | Consumer | United States |
| 173246732 | Omalizumab | Asthma | Enterobiasis | 2020 | Q4 | Consumer | Canada |
| 175066951 | Omalizumab | Unknown | Infection parasitic | 2020 | Q1 | Consumer | United States |
| 175440962 | Omalizumab | Unknown | Parasitic gastroenteritis | 2020 | Q2 | Physician | Uruguay |
| 176897311 | Dupilumab | Unknown | Helminthic infection | 2020 | Q2 | Consumer | United States |
| 177316141 | Benralizumab | Eosinophil Count Increased | Enterobiasis | 2020 | Q2 | Consumer | United States |
| 178661361 | Dupilumab | Unknown | Cestode infection | 2020 | Q2 | Pharmacist | Germany |
| 178773361 | Benralizumab | Asthma | Trichuriasis | 2020 | Q2 | Physician | United States |
| 181342151 | Mepolizumab | Unknown | Helminthic infection | 2020 | Q3 | Consumer | United Kingdom |
| 181359671 | Mepolizumab | Unknown | Helminthic infection | 2020 | Q3 | Consumer | United Kingdom |
| 182472141 | Mepolizumab | Unknown | Infection parasitic | 2020 | Q3 | Consumer | United States |
| 182565491 | Dupilumab | Dermatitis | Enterobiasis | 2020 | Q3 | Consumer | United States |
| 182811391 | Dupilumab | Unknown | Infection parasitic | 2020 | Q3 | Unknown | United States |
| 183252511 | Benralizumab | Unknown | Helminthic infection | 2020 | Q3 | Unknown | United States |
| 183524341 | Dupilumab | Unknown | Helminthic infection | 2020 | Q4 | Consumer | United States |
| 184553801 | Dupilumab | Unknown | Infection parasitic | 2020 | Q4 | Consumer | United States |
| 187547381 | Dupilumab | Unknown | Parasitic gastroenteritis | 2021 | Q1 | Consumer | United States |
| 188900731 | Dupilumab | Unknown | Infection parasitic | 2021 | Q1 | Consumer | United States |
| 189987444 | Dupilumab | Dermatitis | Enterobiasis | 2021 | Q4 | Physician | United States |
| 190275381 | Dupilumab | Unknown | Helminthic infection | 2021 | Q1 | Physician | United States |
| 190772101 | Dupilumab | Unknown | Enterobiasis | 2021 | Q1 | Consumer | United States |
| 191667251 | Dupilumab | Neurodermatitis | Helminthic infection | 2021 | Q2 | Consumer | Germany |
| 192436002 | Dupilumab | Unknown | Infection parasitic | 2021 | Q3 | Consumer | United States |
| 192538592 | Dupilumab | Nasal Polyps | Nematodiasis | 2021 | Q3 | Consumer | United States |
| 194491684 | Dupilumab | Dermatitis Atopic | Helminthic infection | 2021 | Q4 | Physician | United States |
| 194749502 | Dupilumab | Unknown | Infection parasitic | 2021 | Q3 | Consumer | United States |

Table S2 continued

| **Report id** | **Drug** | **Indication** | **Parasitic Infection** | **Report year** | **Quarter** | **Reporter** | **Country** |
| --- | --- | --- | --- | --- | --- | --- | --- |
| 195031201 | Benralizumab | Unknown | Echinococciasis | 2021 | Q3 | Physician | Germany |
| 195420921 | Dupilumab | Unknown | Infection parasitic | 2021 | Q3 | Consumer | United States |
| 196135061 | Mepolizumab | Unknown | Parasitic pneumonia | 2021 | Q3 | Consumer | Austria |
| 196159612 | Dupilumab | Unknown | Infection parasitic | 2021 | Q3 | Consumer | United States |
| 197404401 | Dupilumab | Unknown | Infection parasitic | 2021 | Q3 | Consumer | United States |
| 199016991 | Dupilumab | Unknown | Infection parasitic | 2021 | Q3 | Consumer | United States |
| 199162231 | Dupilumab | Unknown | Infection parasitic | 2021 | Q4 | Physician | United States |
| 199611801 | Dupilumab | Dermatitis Atopic | Helminthic infection | 2021 | Q4 | Physician | United States |
| 201822031 | Dupilumab | Unknown | Enterobiasis | 2021 | Q4 | Physician | United States |
| 201925381 | Dupilumab | Dermatitis Atopic | Helminthic infection | 2021 | Q4 | Physician | United States |

Table S3. Raw data on the occurrence of the parasitic infection, start/end date of the therapy, and calculated time of onset based on available data. Dates are expressed in YYYYMMDD where YYYY=Year, MM=Month, and DD=Day. †From the legacy adverse event reporting system database

| **Report id** | **Drug** | **Date of parasitic infection** | **Start of therapy** | **End date of therapy** | **Time to onset (days)** |
| --- | --- | --- | --- | --- | --- |
| 6412861† | Omalizumab |  |  |  |  |
| 6422338† | Omalizumab |  |  |  |  |
| 7456124† | Omalizumab | 20110406 | 20110330 |  | -24 |
| 7831160† | Omalizumab | 20110522 | 20110413 |  | 9 |
| 88868233 | Omalizumab |  |  |  |  |
| 112279541 | Omalizumab |  |  |  |  |
| 116713721 | Omalizumab |  |  |  |  |
| 120462742 | Omalizumab |  |  |  |  |
| 127270233 | Omalizumab | 20160329 | 20160329 | 20160824 | 0 |
| 128327562 | Mepolizumab | 20160930 | 2016 |  |  |
| 130536502 | Omalizumab | 20161214 | 20160809 |  | 5 |
| 133047542 | Omalizumab | 20160601 | 20160111 |  | -10 |
| 135297952 | Omalizumab | 200910 | 200909 |  |  |
| 135384151 | Omalizumab | 20150526 | 20130730 |  | -4 |
| 135833931 | Omalizumab |  |  |  |  |
| 135951192 | Omalizumab | 201705 | 201705 |  |  |
| 152438235 | Dupilumab | 20180227 | 20161220 | 20161220 | 7 |
| 153768121 | Omalizumab |  |  |  |  |
| 154171474 | Benralizumab | 20180512 | 20180411 |  | 1 |
| 155437031 | Mepolizumab |  |  |  |  |
| 155446756 | Omalizumab | 20180812 | 20180912 |  | 0 |
| 156369229 | Dupilumab | 201901 | 20181115 |  |  |
| 159035971 | Benralizumab |  |  |  |  |
| 159198241 | Omalizumab |  |  |  |  |
| 159279334 | Benralizumab | 201812 | 2018 |  |  |
| 159385621 | Omalizumab | 20181114 | 201807 |  |  |
| 159389361 | Omalizumab |  |  |  |  |
| 160665231 | Mepolizumab |  |  |  |  |
| 160926871 | Benralizumab |  |  |  |  |
| 161733721 | Benralizumab |  |  |  |  |
| 162228012 | Mepolizumab |  |  |  |  |
| 162231351 | Dupilumab |  |  |  |  |
| 163153155 | Dupilumab | 20190426 | 20190426 | 20190426 | 0 |
| 164557632 | Mepolizumab | 201812 | 20160113 |  |  |
| 164821131 | Dupilumab | 201905 | 20170501 |  |  |
| 165259972 | Mepolizumab |  |  |  |  |
| 165380635 | Dupilumab | 2020 | 20190322 | 20201029 |  |
| 165871631 | Dupilumab |  | 20190610 |  |  |
| 166135231 | Omalizumab | 2016 | 201511 | 20160407 |  |

Table S3 continued

| **Report id** | **Drug** | **Date of parasitic infection** | **Start of therapy** | **End date of therapy** | **Time to onset (days)** |
| --- | --- | --- | --- | --- | --- |
| 166854971 | Dupilumab | 20190704 |  |  |  |
| 168228951 | Mepolizumab | 201908 |  |  |  |
| 169536901 | Dupilumab |  |  |  |  |
| 169750711 | Dupilumab | 2019 | 2019 |  |  |
| 170932051 | Mepolizumab |  |  |  |  |
| 173246732 | Omalizumab | 20180227 | 20180227 |  | 0 |
| 175066951 | Omalizumab |  |  |  |  |
| 175440962 | Omalizumab | 20200220 | 201906 |  |  |
| 176897311 | Dupilumab | 202003 | 20200220 |  |  |
| 177316141 | Benralizumab |  | 202001 |  |  |
| 178661361 | Dupilumab |  |  |  |  |
| 178773361 | Benralizumab |  | 20200129 |  |  |
| 181342151 | Mepolizumab |  |  |  |  |
| 181359671 | Mepolizumab |  |  |  |  |
| 182472141 | Mepolizumab | 2020 |  |  |  |
| 182565491 | Dupilumab | 2020 | 202004 |  |  |
| 182811391 | Dupilumab |  |  |  |  |
| 183252511 | Benralizumab | 2020 | 20200824 |  |  |
| 183524341 | Dupilumab | 2020 | 202007 |  |  |
| 184553801 | Dupilumab |  | 20190919 |  |  |
| 187547381 | Dupilumab |  | 20190606 |  |  |
| 188900731 | Dupilumab |  | 20201210 |  |  |
| 189987444 | Dupilumab | 20210212 | 20210212 |  | 0 |
| 190275381 | Dupilumab |  |  |  |  |
| 190772101 | Dupilumab | 20210325 |  |  |  |
| 191667251 | Dupilumab |  | 2021 |  |  |
| 192436002 | Dupilumab | 20210503 | 20180731 |  | -28 |
| 192538592 | Dupilumab | 20210406 | 20200211 | 20210408 | -5 |
| 194491684 | Dupilumab |  | 20210525 | 20211005 |  |
| 194749502 | Dupilumab | 2020 | 2020 |  |  |
| 195031201 | Benralizumab |  |  |  |  |
| 195420921 | Dupilumab | 20210222 | 202011 |  |  |
| 196135061 | Mepolizumab |  |  |  |  |
| 196159612 | Dupilumab |  |  |  |  |
| 197404401 | Dupilumab |  | 202004 |  |  |
| 199016991 | Dupilumab | 20210927 | 20201212 |  | 15 |
| 199162231 | Dupilumab | 20210901 | 202012 |  |  |
| 199611801 | Dupilumab | 20190101 | 20191101 | 20191101 | 0 |
| 201822031 | Dupilumab |  | 20210216 |  |  |
| 201925381 | Dupilumab | 20210101 | 20210525 | 20210525 | -24 |

Table S4. Primary disproportionality analysis, in which “other-drug” represents all other drugs in FAERS. “Event” represents a parasitic infection. Counts A,B,C and D correspond with counts as presented in manuscript Table 1.

| **Drug** | **ATC** | **Drug-event count (A)** | **Drug-other event count (B)** | **Other drug-event count (C)** | **Other drug-other event count (D)** | **ROR**  **(95% CI)** |
| --- | --- | --- | --- | --- | --- | --- |
| Omalizumab | R03DX05 | 25 | 149,272 | 1,597 | 37,368,549 | 3.9  (2.6 - 5.8) |
| Dupilumab | D11AH05 | 34 | 201,181 | 1,588 | 37,316,640 | 4.0  (2.8 - 5.6) |
| Mepolizumab | R03DX09 | 12 | 47,270 | 1,610 | 37,470,551 | 5.9  (3.4 - 10.4) |
| Benralizumab | R03DX10 | 10 | 14,807 | 1,612 | 37,503,014 | 15.7  (8.4 - 29.3) |
| Reslizumab | R03DX08 | 0 | 997 | 1,622 | 37,516,824 | NA |

ATC = Anatomical Therapeutic Chemical classification, NA = Not Applicable, ROR= Reporting Odds Ratio, 95% CI = 95% Confidence Interval

Table S5. 1^st^ secondary disproportionality analysis, in which “other-drug” represents all other drugs in FAERS, excluding all drugs under ATC level 5 “P”. “Event” represents a parasitic infection. Counts A,B,C† and D† correspond with counts as presented in manuscript Table 1.

| **Drug** | **ATC** | **Drug-event count (A)** | **Drug-other event count (B)** | **Other drug-event count (C†)** | **Other drug-other event count (D†)** | **ROR**  **(95% CI)** |
| --- | --- | --- | --- | --- | --- | --- |
| Omalizumab | R03DX05 | 25 | 149,272 | 1,443 | 37,267,467 | 4.3  (2.9 - 6.4) |
| Dupilumab | D11AH05 | 34 | 201,181 | 1,434 | 37,215,558 | 4.4  (3.1 - 6.2) |
| Mepolizumab | R03DX09 | 12 | 47,270 | 1,456 | 37,369,469 | 6.5  (3.7 - 11.5) |
| Benralizumab | R03DX10 | 10 | 14,807 | 1,458 | 37,401,932 | 17.3  (9.3 - 32.3) |
| Reslizumab | R03DX08 | 0 | 997 | 1,468 | 37,415,742 | NA |

ATC = Anatomical Therapeutic Chemical classification, NA = Not Applicable, ATC code “P” = Antiparasitic products, insecticides and repellents, ROR= Reporting Odds Ratio, 95% CI = 95% Confidence Interval

Table S6. 2^nd^ secondary disproportionality analysis, in which “other-drug” represents the other biologics in this table. “Event” represents a parasitic infection. Counts A,B,C‡ and D‡ correspond with counts as presented in manuscript Table 1.

| **Drug** | **ATC** | **Drug-event count (A)** | **Drug-other event count (B)** | **Other drug-event count (C**‡**)** | **Other drug-other event count (D**‡**)** | **ROR**  **(95% CI)** |
| --- | --- | --- | --- | --- | --- | --- |
| Dupilumab | D11AH05 | 34 | 201,181 | 47 | 212,346 | 0.8  (0.5 - 1.2) |
| Omalizumab | R03DX05 | 25 | 149,272 | 56 | 264,255 | 0.8  (0.5 - 1.3) |
| Mepolizumab | R03DX09 | 12 | 47,270 | 69 | 366,257 | 1.4  (0.7 - 2.5) |
| Benralizumab | R03DX10 | 10 | 14,807 | 71 | 398,720 | 3.8  (2.0 - 7.4) |
| Reslizumab | R03DX08 | 0 | 997 | 81 | 412,530 | NA |

ATC = Anatomical Therapeutic Chemical classification, NA = Not Applicable, ROR= Reporting Odds Ratio, 95% CI = 95% Confidence Interval
